# A Misclassification of Pulmonary Stenosis Using Conventional Echocardiographic Methods

**DOI:** 10.4274/balkanmedj.2016.0903

**Published:** 2018-01-20

**Authors:** Tuğba Kemaloğlu Öz, Mehmet Eren, Tayfun Gürol, Özer Soylu, Bahadır Dağdeviren

**Affiliations:** 1 Clinic of Cardiology, Dr. Siyami Ersek Thoracic and Cardiovascular Surgery Training and Research Hospital, İstanbul, Turkey; 2 Department of Cardiology, Bahçeşehir University School of Medicine, İstanbul, Turkey

**Keywords:** Atrial septal defect, echocardiography, pregnancy, pulmonary stenosis, three-dimensional echocardiography

## Abstract

**Background:**

The classification of pulmonary stenosis (PS) severity based on the transpulmonary pressure gradient, which is affected by flow rate.

**Case Report:**

We report the first case of a pregnant patient with atrial septal defect (ASD) and pulmonary stenosis that was misclassified by conventional echocardiographic methods. Most importantly, three-dimensional transoesophageal echocardiographic assessment of pulmonary stenosis changed the entire treatment strategy.

**Conclusion:**

The planimetric calculation of the pulmonary valve (PV) opening area using three-dimensional transoesophageal echocardiographic may be helpful, especially in encounters with specialized conditions such as ASD and/or pregnancy, which can cause inaccurate recordings of the transvalvular peak gradient.

Atrial septal defects (ASDs) are common in women and, depending on their size, can at present any age. In most instances, ASDs are diagnosed during pregnancy (1). The most common pathology of the pulmonary valve (PV) is pulmonary stenosis (PS), which is seen in almost 10% of adult patients with congenital heart disease (2). The first step in the evaluation of PS involves echocardiography. The most commonly used technique for measuring the severity of PS is Doppler imaging (3); however, unlike other valves (e.g. aortic valve), there is little information concerning the PV. PS severity is classified according to the transpulmonary pressure gradient and maximal velocity. The PV area cannot be calculated by planimetry using two-dimensional echocardiography because the *en face* view of PV is generally unavailable (4). Here, we report the first case of a pregnant patient with ASD and PS who was misdiagnosed by conventional echocardiographic methods. A three-dimensional (3D) transoesophageal echocardiographic assessment of the PS changed the treatment strategy for this patient.

## CASE PRESENTATION

**Figure 1:**
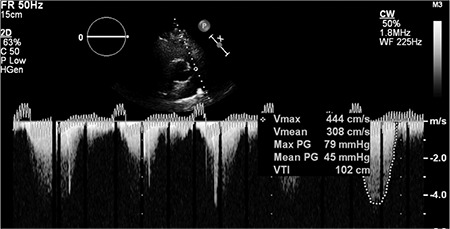
Continuous wave Doppler of the pulmonary valve.

**Figure 2:**
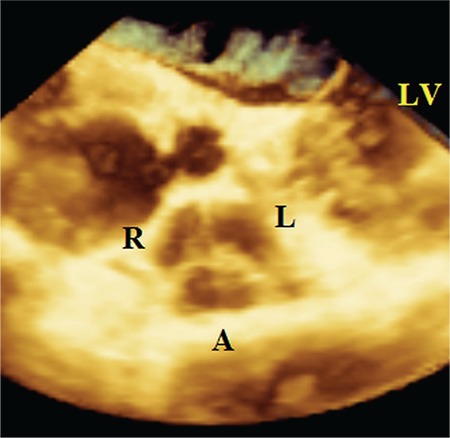
A real-time three-dimensional transoesophageal echocardiography that demonstrates an en face view of a tricuspid pulmonary valve that was imaged from the pulmonary artery in diastole. *A: anterior leaflet; L: left leaflet; LV: left ventricle; R: right leaflet*

**Figure 3:**
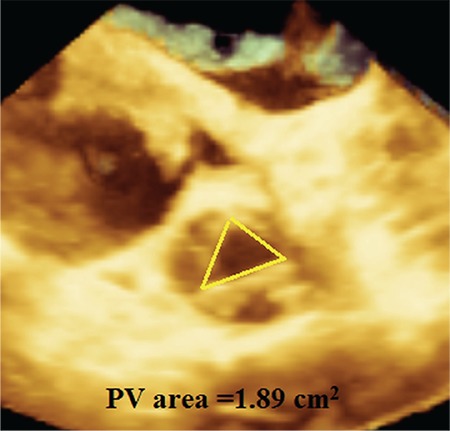
A real-time three-dimensional transoesophageal echocardiography, an en face view of the tricuspid pulmonary valve imaged from the pulmonary artery in systole. The yellow triangle shows the pulmonary valve opening area. *PV: pulmonary valve*

A 20-year-old pregnant woman with secundum ASD and severe PS presented with progressive dyspnoea and was referred to our clinic for a percutaneous ASD closure and a pulmonary valvuloplasty. She was in the second trimester (16 weeks) of pregnancy. At admission, her arterial blood pressure and heart rate were 120/70 mmHg and 64 beats/min, respectively. The physical exam was unremarkable except for a grade 2/6 ejection systolic murmur best heard over the left second intercostal space and fixed splitting of the second heart sound. An electrocardiogram showed an incomplete right bundle branch block. A two-dimensional transthoracic echocardiography (2D TTE) was done using a Philips (Bothell, WA, USA) iE33 ultrasound system with an x5-1 transducer. It showed a secundum ASD with a left-to-right shunt, severe PS (maximal gradient= 79 mmHg and maximal velocity= 4.4 m/s) (Figure 1), an enlarged right ventricle (RV) (basal diameter= 4.4 cm), normal left ventricular function and mild tricuspid regurgitation. The RV free wall was 0.4 cm. Tricuspid annular plane systolic excursion were measured at 1.5 cm. Then, 2D transoesophageal echocardiography (TEE) was done using the X7-2t matrix transducer and the same ultrasound system as the 2D TTE. The 2D TEE findings were the same as those of 2D TTE, but the three leaflets of the PV were still not visualized; however, there were thickening of leaflets and a doming motion. For further evaluation, we performed a 3D TEE using the same system and transducer. Full-volume data of the PV and inter-atrial septum were taken, and all images were systematically and sequentially cropped to view both the ASD and PV *en face* in both non-multiplanar reconstruction (MPR) and MPR modes. The secundum ASD was measured 2.4 x 1.6 cm with an area of 4.5 cm^2^ and all rims were larger than 5 mm. Thus, the ASD was determined to be appropriate for a percutaneous closure procedure. The PV was noted to be tricuspid (Figure 2) and the PV area was 1.89 cm^2^ (Figure 3). PS was classified according to the planimetric method as a mild stenosis (5,6).

There was an inconsistency between the Doppler and 3D classification of PS severity. During pregnancy, ESC guidelines on the management of cardiovascular diseases during pregnancy (7) provide follow-up recommendations for patients with mild or moderate PS, but balloon valvuloplasty should be advised for symptomatic severe PS with RV dysfunction as in our patient. Therefore, we decided to follow up our patient during pregnancy. After the pregnancy was completed, the peak PV gradient was reduced to 41 mmHg (a moderate PS according to the gradient/velocity-based classification). The patient then underwent a percutaneous closure of the ASD. After a successful procedure, the peak PV gradient reduced to 26 mmHg (a mild PS according to the gradient/velocity-based classification), and the discrepancy between the Doppler and planimetry-based classification for PS was eliminated. Written informed consent was obtained from the patient.

## DISCUSSION

Pregnancy results in profound physiologic changes. Chief among these changes are increases in cardiac output, heart rate, blood volume, biventricular stroke work and oxygen consumption. Also, women with congenital heart disease may not tolerate these hemodynamical changes very well. However, most women with unrepaired ASDs have successful pregnancies (8). In addition, ASDs can cause left-to-right shunts and volume overload of the right atrium and RV. In our patient, the maximal PV gradient was overestimated for these two reasons. We know the planimetric method is not affected by flow rate (4); thus, it is more useful than gradient- and velocity-based classifications, especially in situations such as these.

We were unable to provide a correct treatment decision based only on symptoms such as dyspnoea, palpitation or chest pain because these symptoms are common among pregnant women. 

Symptom- and Doppler-based misclassifications of PS may influence the accuracy of the valvuloplasty time and may lead to a termination of the pregnancy due to unnecessary radiation exposure. Therefore, the 3D TEE planimetric method is useful and safe, especially in the RV volume overloading period when the transvalvular peak gradient of the PV is affected. The planimetric calculation of the PV opening area using 3D TEE may be helpful, especially when specialized conditions such as ASD or/and pregnancy can cause inaccurate recordings of the transvalvular peak gradient and maximal velocity.
